# Canine Mammary Tumours Are Affected by Frequent Copy Number Aberrations, including Amplification of *MYC* and Loss of *PTEN*

**DOI:** 10.1371/journal.pone.0126371

**Published:** 2015-05-08

**Authors:** Kaja S. Borge, Silje Nord, Peter Van Loo, Ole C. Lingjærde, Gjermund Gunnes, Grethe I. G. Alnæs, Hiroko K. Solvang, Torben Lüders, Vessela N. Kristensen, Anne-Lise Børresen-Dale, Frode Lingaas

**Affiliations:** 1 Section of Genetics, Department of Basic Sciences and Aquatic Medicine, Faculty of Veterinary Medicine and Biosciences, Norwegian University of Life Sciences (NMBU),Oslo, Norway; 2 Department of Genetics, Institute for Cancer Research, Division of Cancer Medicine, Surgery and Transplantation, Oslo University Hospital Radiumhospitalet, Oslo, Norway; 3 Wellcome Trust Sanger Institute, Hinxton, Cambridge, United Kingdom; 4 Human Genome Laboratory, Department of Human Genetics, VIB and University of Leuven, Leuven, Belgium; 5 Biomedical Informatics, Department of Informatics, University of Oslo, Oslo, Norway; 6 Centre for Cancer Biomedicine, University of Oslo, Oslo, Norway; 7 Section of Anatomy and Pathology, Department of Basic Sciences and Aquatic Medicine, Faculty of Veterinary Medicine and Biosciences, Norwegian University of Life Sciences (NMBU), Oslo, Norway; 8 Marine Mammals Research Group, Institute of Marine Research, Bergen, Norway; 9 Institute for Clinical Medicine, Faculty of Medicine, University of Oslo, Oslo, Norway; 10 Department of Clinical Molecular Biology and Laboratory Sciences (EpiGen), Akershus University Hospital, Lørenskog, Norway; 11 The K. G. Jebsen Center for Breast Cancer Research, Institute for Clinical Medicine, Faculty of Medicine, University of Oslo, Oslo, Norway; 12 Department of Clinical Molecular Biology, Division of Medicine, Akershus University Hospital, Ahus, Norway; University of Sydney, AUSTRALIA

## Abstract

**Background:**

Copy number aberrations frequently occur during the development of many cancers. Such events affect dosage of involved genes and may cause further genomic instability and progression of cancer. In this survey, canine SNP microarrays were used to study 117 canine mammary tumours from 69 dogs.

**Results:**

We found a high occurrence of copy number aberrations in canine mammary tumours, losses being more frequent than gains. Increased frequency of aberrations and loss of heterozygosity were positively correlated with increased malignancy in terms of histopathological diagnosis. One of the most highly recurrently amplified regions harbored the *MYC* gene. *PTEN* was located to a frequently lost region and also homozygously deleted in five tumours. Thus, deregulation of these genes due to copy number aberrations appears to be an important event in canine mammary tumour development. Other potential contributors to canine mammary tumour pathogenesis are *COL9A3*, *INPP5A*, *CYP2E1* and *RB1*. The present study also shows that a more detailed analysis of chromosomal aberrations associated with histopathological parameters may aid in identifying specific genes associated with canine mammary tumour progression.

**Conclusions:**

The high frequency of copy number aberrations is a prominent feature of canine mammary tumours as seen in other canine and human cancers. Our findings share several features with corresponding studies in human breast tumours and strengthen the dog as a suitable model organism for this disease.

## Background

Cancer is the most frequent cause of disease-associated death in dogs, and naturally occurring cancer is well described in several breeds [1,2]. Canine mammary tumours (CMTs), which are among the most common canine cancer forms [3], are often seen in elderly, intact bitches [4,5]. A higher incidence in certain breeds such as the English Springer Spaniel, Boxer, Cocker Spaniel and Dachshund [4–9] strongly suggests an underlying genetic predisposition. However, CMT-associated genetic risk factors largely remain to be identified. Comparing canine and human mammary cancers, the latter are dominated by epithelial tumours, while CMTs frequently also contain myoepithelial and mesenchymal components [10]. Further, regional lymph node metastasis has been reported to be a less important prognostic factor in dogs than in humans [11]. Nevertheless, canine and human mammary cancers share important clinical and pathophysiologic characteristics: a spontaneous occurrence of tumours that primarily affect females, hormonal influence on tumour development (e.g. oestrogen and progesterone), histopathological similarities, broadly equal metastatic spread, and potentially also mutual prognostic markers and genetic risk factors for disease [10,11].

Since their domestication from the wolf, dogs have continuously undergone artificial selection [12–14]. Most breeds are descended from a small number of founders and popular sires [15]. As a result, genetic isolated populations of breeds have developed, many at increased risk of specific genetic disorders [13,16–19]. The breed structure of the dog with low genetic heterogeneity within breeds and long linkage disequilibrium (LD), combined with the enrichment of specific disease-associated alleles within certain breeds, is an advantage in mapping/identification of genes of interest for genetic disorders.

Cancer genomes are typically characterized by numerous sequence changes compared with their normal host counterparts. An important example of such changes is the accumulation of copy number alterations, a key event in the development and progression of many cancers. Such alterations range from single nucleotide insertions or deletions to gain or loss of large chromosomal fragments, and even whole-genome duplications [20,21]. Detection of recurrent DNA copy number alterations (CNAs) and identification of the genes affected by such alterations can reveal underlying mechanisms of disease evolution and might be of diagnostic, prognostic and therapeutic significance. Human breast cancer is a heterogeneous disease with established subtypes based on gene expression profiling [22,23]. Distinct spectra of CNAs have been shown to underlie each of these subtypes. This strongly suggests that tumours of different subtypes develop along specific genetic pathways [24–28] and that different mechanisms of genomic instability give rise to the different breast tumours subtypes and may contribute to their characteristic biological and clinical behavior [26]. Genetic subtypes of CMTs based on expression analysis and CNAs remain to be defined, although immunohistochemical analysis indicates that some of the same subgroups can be found in canine mammary neoplasms [29,30]. Further, previous studies have demonstrated that CNAs in a variety of human cancers are recurrently observed in the corresponding canine cancer [31–33]. A recent study of genome aberrations in canine mammary carcinomas also reported similarities to human breast cancer as well as specific canine alterations [34]. Defining aberrant genomic regions in canine tumours and subsequent comparative molecular analysis in human provides an opportunity to gain deeper insight into pathways implicated in mammary tumourigenesis in both species and to reveal additional regions not yet identified in human breast cancer. It may also be an important step in the work towards developing new cancer treatments.

In the present survey, we aimed to identify regions and genes associated with canine mammary tumour initiation and progression. To our knowledge, this is the first study of allele-specific copy number in canine mammary tumours. Significance analysis of the aberrant regions was also performed.

## Materials and Methods

### Ethics Statement

The blood and tumour samples of the present study were collected by certified veterinarians at private veterinary clinics in Norway during routine clinical and mammary tumour surgery procedures, and in agreement with the provisions enforced by the Norwegian Animal Research Authority and the Norwegian Regulation on Animal Experimentation [35].

### Samples

DNA from blood and mammary tumour samples from 69 dogs of different breeds were included in the study. These were privately owned dogs presented to a veterinarian due to the occurrence of mammary tumours. From 46 of the dogs we had DNA from multiple tumours (44 with two and two with three tumours) and 23 dogs were represented by one tumour, in total 117 tumours. Two biopsies of maximum 0.5cm^3^ were taken next to each other from the tumours that had been surgically removed from the dogs. One biopsy was submerged in formalin and the other in RNA*later* (Ambion, Applied Biosystems, Austin, Texas, USA). All the biopsies were mailed to the Norwegian School of Veterinary Science (NSVS), and the RNAlater biopsies were frozen at -80 degrees at arrival. The formalin biopsies were paraffin-embedded, sectioned by microtome and stained (hematoxylin-eosin and van Gieson). All the tumour sections were examined and classified by one pathologist (GG) according to the latest proposal for WHO guidelines for CMTs [36] (Gjermund Gunnes, Kaja S. Borge, Frode Lingaas; submitted). Each tumour sample was examined for tumour size, growth pattern, secondary changes, cell and tissue types, nucleus, and invasive growth into tumour stroma. Tumor size was scored from 1 to 3, where 1: tumours <1 cm in diameter 2: tumours between 1 and 2.9 cm in diameter, and 3: tumours >3 cm in diameter. Growth pattern included hyperplasia and solid growth of the epithelial component. It was evaluated from 1 to 3, where 0: not observed, 1: < 25% of section area, 2: 25–90% of section area, and 3: > 90% of section area. Secondary changes like connective tissue, inflammation and necrosis was evaluated from 0 to 3, where 0: not observed, 1: < 10% of section area, 2: 10–75% of section area, and 3: > 75% of section area. The nucleus groups included nuclear pleomorphism, mitotic index and mitotic morphology. All three parameters were scored from 0 to 3. For nuclear pleomorphism, 0: no irregular nuclei, 1: small degree of variation in nuclear shape and size, and occasional nucleoli, 2: moderate degree of variation in nuclear shape and size, and presence of nucleoli, and 3: high degree of variation in nuclear shape and size, and numerous prominent nucleoli. For mitotic index, 0: no observed mitotic figures in 10 HPF, 1: fewer than 10 mitotic figures per 10 HPF, 2: 10 to 19 mitotic figures per 10 HPF, and 3: 20 or more mitotic figures per 10 HPFF. For mitotic morphology (number of mitoses with abnormal mitotic morphology), 0: none, 1: few, 2: moderate amounts, 3: numerous. The parameters in the cell and tissue type group (epithelial cells, myoepithelial cells, chondroid tissue, osteoid tissue) and invasive growth into tumour stroma were scored on a binomial scale where 0: not observed and 1: observed. The most common benign and malignant diagnosis in the present material of 117 tumours were complex adenoma (adenomyoepithelioma) (n = 26) and complex carcinoma (n = 17), respectively. A method for simultaneous extraction of RNA and DNA from small biopsies was applied to the RNAlater-tumour biopsies. RNA was first isolated according to the protocol described by Riis et al. [37]. Thereafter, DNA was extracted from the interphase/organic phase left after RNA isolation through the following steps: Back extraction buffer [38] was used to separate the phases, isopropanol added to precipitate DNA, and the precipitated DNA transferred to DNeasy Mini columns (Qiagen, Hilden, Germany). Further processing was performed according to Qiagen’s protocol, and the purified DNA was eluted with water. DNA quantity and quality was measured by NanoDrop (Thermo Fisher Scientific, Wilmington, Pennsylvania, USA) and diluted to a concentration of 50ng/μl. The DNA was used for the array analysis of the present study, while the RNA was saved at -80 degrees for use in future analysis.

### Array analysis

Illumina CanineHD Genotyping BeadChips, 170K (Illumina, Inc., San Diego, CA, USA), were used for analysis of the blood and tumour samples. The hybridized arrays were scanned by Illumina BeadArray Reader and the scanning results imported into GenomeStudio Genotyping Module. All samples were custom clustered in GenomeStudio using the blood samples as reference. Estimated B allele frequencies (BAF) and Log R Ratios (LRR) for both blood and tumour samples were used for further analysis (original BAF and LRR values are publically available in the Dryad repository: doi:10.5061/dryad.7dm4d [39,40]).

### Copy number and significance analysis

Tumour LRR values frequently display some bias that can be related to local variations in the GC content and which appears visually as a waving pattern when LRR is plotted against genomic location [41]. As this bias can distort the statistical analysis, the GC correction method implemented in PennCNV [41] was first applied to the data. Next, the arrays were quantile normalized to a normal reference distribution to reduce the effect of technical artifacts that manifest themselves as skewness in the LRR distribution of many arrays (GC corrected, quantile normalized LRR values are available in the Dryad repository: doi:10.5061/dryad.7dm4d [39,40]). The subsequent analysis considered all tumours in the material collectively, as well as different subgroupings: (1) three subgroups defined as hyperplasias (n = 13, including two duct ectasias and two fibroadenomatous changes), benign tumours (n = 57) and malignant tumours (n = 47); (2) groups of morphologically similar diagnosis (Table 1); and (3) six histopathological parameters of malignancy.

**Table 1:** Groups of morphologically related diagnosis used for analysis.

Diagnosis group	Tumour diagnoses included	N
Non-neoplastic tumours	Hyperplasias, ectasias and other non-neoplastic diagnoses of the mammary tissue	12
Benign neoplasias	Simple adenomas (incl. basaloid and intraductal papillary adenomas), complex adenomas, benign mixed tumours	55
Transitional tumours	Carcinoma in situ, carcinoma arising in benign tumor	6
Malignant epithelial tumours with myoepithelial and mesenchymal components	Complex carcinomas and mixed carcinomas, carcinosarcoma, carcinoma and malignant myoepithelioma	18
Malignant purely epithelial with tubular formations	Simple carcinomas (tubular, tubulopapillary, cystic-papillary, cribriform) and ductal carcinoma	18
Malignant purely epithelial with loss of tubular formation and anaplasia	Carcinoma-micropapillary—invasive, solid carcinoma, comedocarcinoma, anaplastic carcinomas	4

N = Number of tumours.

To evaluate genome-wide gains and losses, we first segmented the data using the Piecewise Constant Fit (PCF) algorithm in the Bioconductor package *Copynumber* [42] with penalty parameter gamma = 80 and at least kmin = 10 probes per segment. Following the recommendations of the package, the segmentation was preceded by Winsorization (also available in *Copynumber*) to handle extreme outliers. As the C*opynumber* package is adapted to human data with 23 chromosomes, the canine data were split in two sets (canine chromosome (CFA) 1–19 and CFA20-38 + X) for winsorization and segmentation and were subsequently joined before further frequency analysis of gains and losses. Aberrations were defined as segmented copy number values above 0.05 (gain) or below -0.05 (loss). Regions with aberrations occurring in ≥20% of the tumours were defined as recurrent and further analyzed and compared to the UCSC reference gene list for CanFam 2.0 [43] to identify gained and lost genes. The gained/lost gene lists were compared to the Cancer Gene Census working list of known cancer genes in humans [44]. Similar analysis was performed for the three subgroups of hyperplasias, benign and malignant tumours.

To further investigate the structure of the copy number alterations, we applied the Allele-Specific Copy Number Analysis of Tumours (ASCAT) [24,45]. ASCAT was performed according to Van Loo, Nordgard et al. [24] to estimate tumor purity and ploidy and dissect the allele-specific copy number of the CMTs. Genome-wide ASCAT profiles were calculated for each tumour. Germline BAF, germline LRR, tumour BAF and GC corrected, quantile normalized tumour LRR was used as input data for the ASCAT analysis (ASPCF penalty parameter = 0.80, ASCAT gamma = 0.40, ASCAT version 2.2). Based on the ASCAT output, frequency of gains, losses and loss of heterozygosity (LOH) were calculated as described by Van Loo, Nordgard et al. [24]. Regions with aberrations occurring in ≥20 tumours were defined as recurrent and further analyzed and compared to the UCSC reference gene list for CanFam 2.0 [43] to identify gained and lost genes. UCSC Batch Coordinate Conversion (liftOver) [46] and Ensembl [47] was used to analyze orthology to the human genome. Analysis of ploidy and aberrant cell fraction were performed in R [48] for different malignancy subgroups (hyperplasias, benign and malignant tumours) and groups of tumours of morphological similar diagnosis (Table 1). In addition, the pattern of CNAs was studied at the chromosome level for the following histopatholgical parameters: solid growth of the epithelial component in the tumour (no/yes), presence of myoepithelial cells in the tumour (no/yes), invasive growth of neoplastic cells into tumour stroma (no/yes), nuclear pleomorphism (no/moderate-severe), mitotic index (0/above 9 per 10 high-power fields (HPF)) and presence of necrosis in tumour (no/yes). Solid growth (lack of tubular formation), invasive growth into tumour stroma, increased nuclear pleomorphism and mitotic index, presence of necrosis and absence of myoepithelial cell proliferation are all associated with malignancy and/or poor prognosis in mammary tumours [49–51]. These parameters were part of a larger morphological classification system used to evaluate and diagnose the CMTs of the present study (Gjemund Gunnes, Kaja S. Borge, Frode Lingaas; submitted) (histopathomorphological scoring values for these six parameters for all tumours with an ASCAT output are available in the DRYAD repository: doi:10.5061/dryad.7dm4d [39,40]). The difference between the subgroups of each parameter (no/yes, no/moderate-severe, 0/above 9 pr. 10 HPF) was calculated by subtracting the aberration frequency of the first subgroup from that of the second. An exception was made for the myoepithelial cell presence, where the CNA frequency of the second group (“yes”) was subtracted from the first (“no”). Chromosome regions with such a difference in aberration frequency of ≥20% were registered and further studied.

To investigate the significant peak regions for amplification or deletion, i.e. regions that are more likely to contain “driver mutations” for CMTs, the original idea of the numerical algorithm called GISTIC (Genomic Identification for Significant Targets [52]) was applied to the data. However, since GISTIC has been developed to identify broad/focal regions of the genome that are significantly amplified or deleted across a set of human samples, it was difficult to adapt to our canine data. Therefore, the basic numerical algorithm of GISTIC was applied to the GC corrected, quantile normalized tumour LRR values in this study. The calculation procedure is summarized as following: (1) Take the sum of the segmented data across all samples for each genomic location, (2) Generate permutated data across genomic locations and apply the same procedure 1 to the data for each genomic location, (3) Repeat 2 for 1000 times to generate the null distribution, (4) Find the location satisfied |the value by 1| > the value by 3 for each genomic location and calculate the p-values, and (5) Apply FDR [53] (e.g.5%) for the p-values obtained by 4 and find the significant genomic location. For these analysis purposes, the synchronous tumour pairs from the same dog were split into two different data sets since tumours from the same dog possibly are biologically related and thus not statistically independent observations. The single tumours were included in both sets. The analysis was also performed for a third data set of all tumours together (both single and all the paired tumours). The results from the independent analyses of all three data sets were compared

## Results

### Recurrent gains and losses identified by segmentations

Several regions of gain and loss were identified by the PCF segmentation analysis. In the recurrently gained regions, 80 genes were identified (S1 Table), of which seven were found in the Cancer Gene Census list [44] (Table 2). These seven were all genes with dominant effect, including well-known oncogenes like *FGFR2* and *MYC*. The recurrently lost regions contained 482 genes (S2 Table) of which six were tumour suppressor genes also found in the Cancer Gene Census list (Table 2). The frequency of aberrations increased with malignancy; the number of recurrently gained/lost region was lowest in hyperplasias and highest in malignant tumours (data not shown). Genes from the Cancer Gene Census list that were found in the recurrently aberrant regions for hyperplasias, benign and malignant tumours are presented in Table 3.

**Table 2:** Genes with known cancer-association in humans found in recurrently gained/lost regions in CMTs.

Gene	Human chromosome	Canine chromosome	Genetic effects	Aberration in CMT analysis
*BCL6*	3	34	Dominant	Gain
*FGFR2*	10	28	Dominant	Gain
*FHIT*	3	20	Dominant	Gain
*MITF*	3	20	Dominant	Gain
*MYC*	8	13	Dominant	Gain
*NPM1*	5	4	Dominant	Gain
*PDGFRB*	5	4	Dominant	Gain
*BMPR1A*	10	4	Recessive	Loss
*KDM5C*	X	X	Recessive	Loss
*KDM6A*	X	X	Recessive	Loss
*MEN1*	11	18	Recessive	Loss
*PRF1*	10	4	Recessive	Loss
*SDHB*	1	2	Recessive	Loss

Results from the PCF analysis. The Cancer Gene Census List was used as reference for genes with known cancer-association in humans [44].

**Table 3:** Genes with known cancer-association in humans found in gained/lost regions in different subgroups of CMTs.

Group of tumours	Gained genes	Lost genes
Hyperplasias	*FHIT*, *MITF*, *MYC*, *NPM1*	*AKT2*, *BIRC3*, *BMPR1A*, *CCND1*, *GNA11*, *KDM5C*, *KLK2*, *MEN1*, *MYH9*, *PDGFB*, *PPARG*, *PRF1*, *SDHB*, *TFRC*, *ZNF331*
Benign tumours	*FGFR2*, *FHIT*, *MITF*, *MYC*, *NPM1*, *PDGFRB*	*AKT2*, *BMPR1A*, *CCND1*, *GNA11*, *HMGA1*, *KDM5C*, *KDM6A*, *KLK2*, *MEN1*, *MYH9*, *PDGFB*, *PIM1*, *PPARG*, *PRF1*, *SDHB*, *TFRC*, *ZNF331*
Malignant tumours	*BCL2*, *BCL6*, *CTNNB1*, *FGFR2*, *FHIT*, *GNAQ*, *GNAS*, *KDR*, *KIT*, *LIFR*, *MET*, *MITF*, *MYC*, *NPM1*, *PDGFRB*	*AKT2*, *BIRC3*, *BMPR1A*, *BRCA1*, *CCND1*, *COL1A1*, *ERBB2*, *GNA11*, *HMGA1*, *KDM5C*, *KDM6A*, *KLK2*, *MEN1*, *MUC1*, *MYH9*, *PDGFB*, *PIM1*, *PRF1*, *PTEN*, *RARA*, *SDHB*, *TFRC*, *ZNF331*

Results from the PCF analysis. Genes with recessive molecular genetic function are underlined, otherwise dominant genes [44].

### ASCAT

ASCAT profiles were obtained for 113 tumours, of which 12 were hyperplasias/ectasias, 55 benign and 46 malignant neoplasias.

#### Ploidy

**Fig 1:**
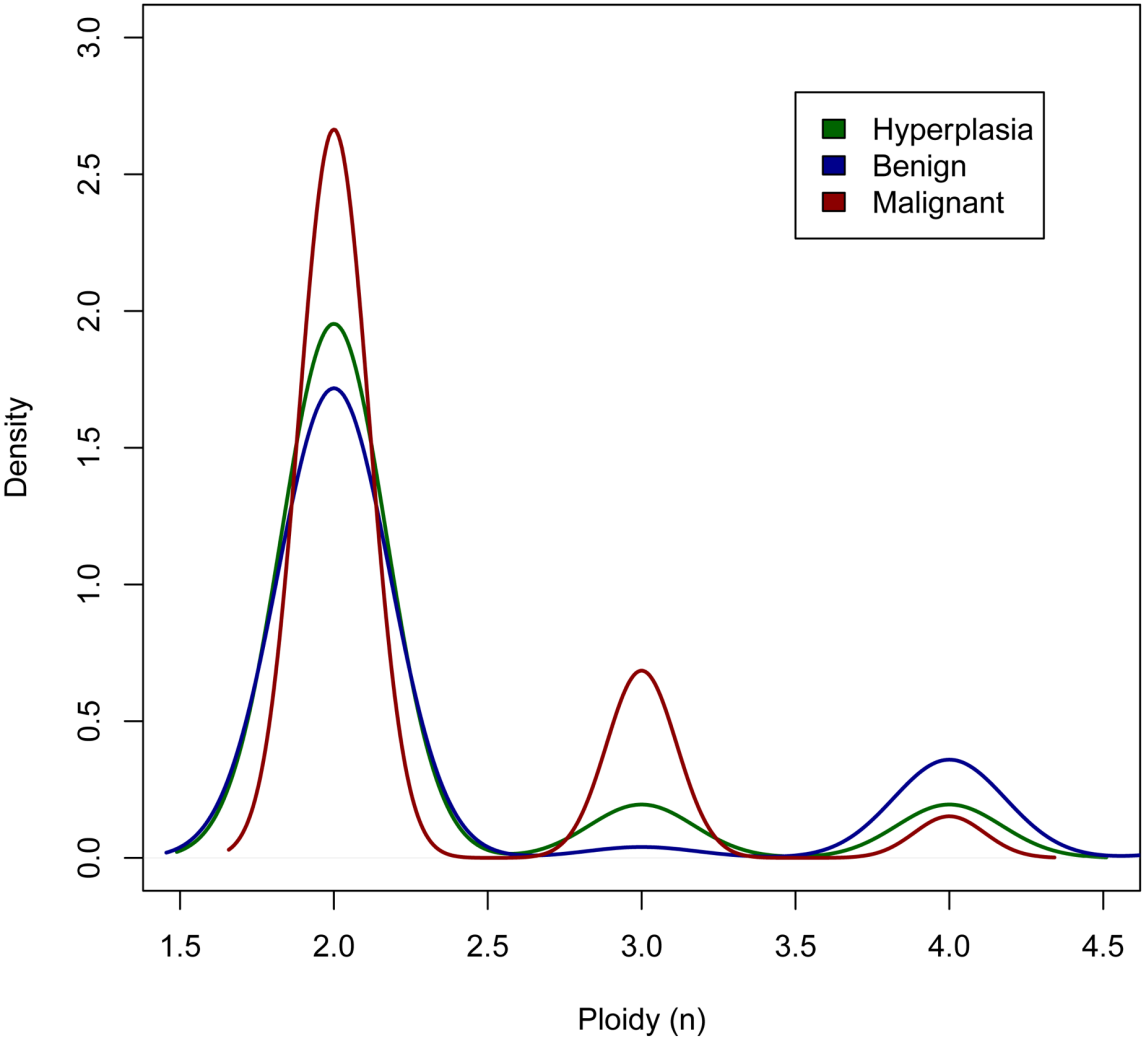
ASCAT estimate of ploidy in different subgroups of tumours. Hyperplasias (n = 12), benign (n = 55) and malignant tumours (n = 46). For the graphical presentation, the ploidy estimates were rounded to the nearest whole number.

A mean ASCAT ploidy estimate of 2.4 was found across all tumours. Eighty-four (~74%) of the tumours were diploid (ploidy close to 2; 1.8–2.2) and 29 (~26%) were aneuploid (ploidy <1.8 or >2.2). Of the aneuploid tumours, two (~2%) were hypodiploid (ploidy <1.8), ten (~9%) tetraploid (ploidy close to 4; 3.8–4.2) and two (~2%) highly polyploid (ploidy >4.2). The proportion of the hyperplasias, benign and malignant tumours with aneuploidy was ~17%, 26% and 28%, respectively. Tumours of a benign CMT diagnosis most often had a ploidy estimate of either ~2 or ~4, while a substantial part of the malignant tumours had a ploidy of ~3 (Fig 1).

#### Aberrant cell fraction

**Fig 2:**
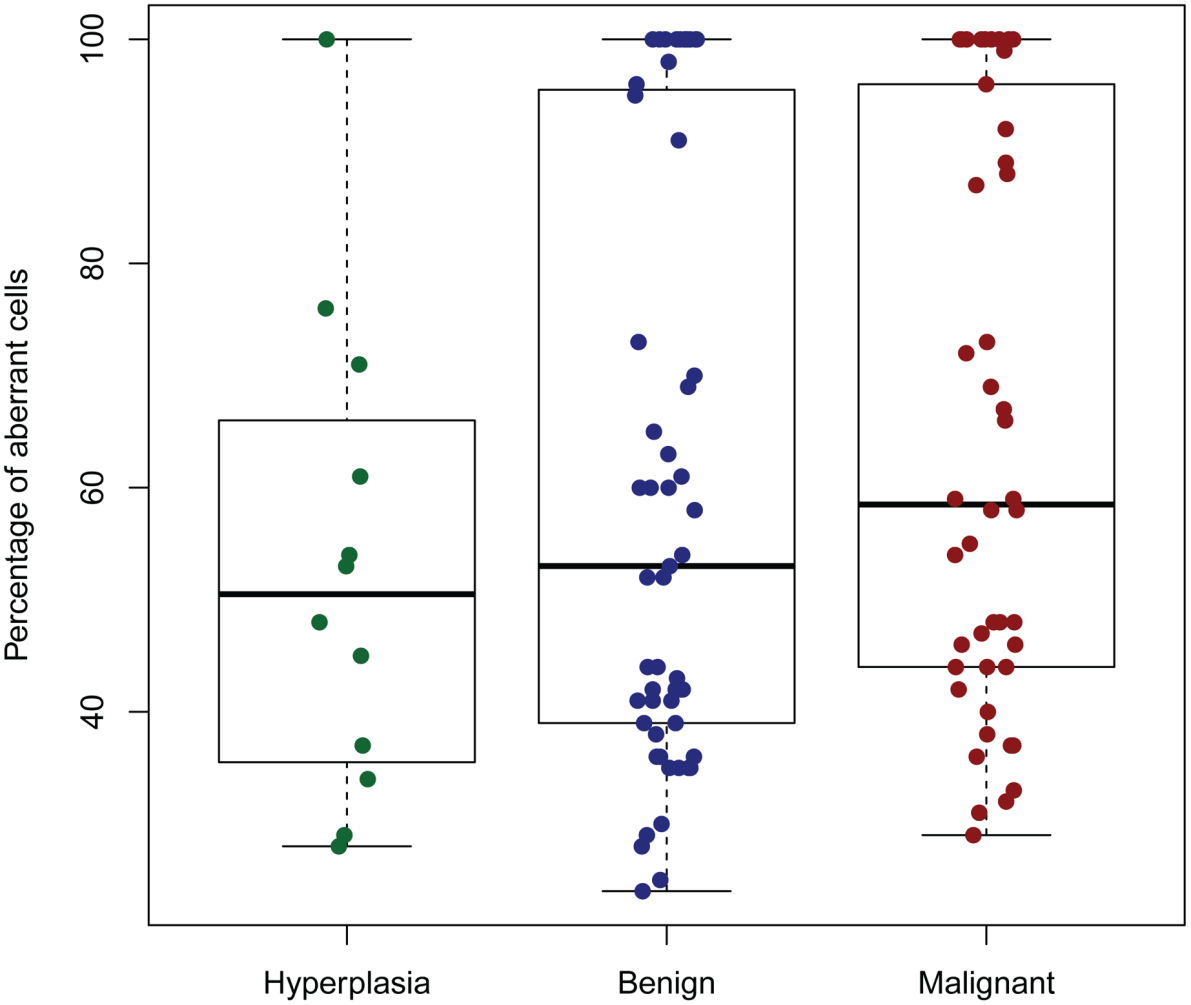
ASCAT estimate of percentage of aberrant cells in different subtypes of tumours. Hyperplasias (n = 12), benign tumours (n = 55) and malignant tumours (n = 46).

The estimated aberrant cell fraction (percentage of tumour cells) from the ASCAT profiles ranged from 24% to 100%, with a mean of 62%. In general, malignant tumours tended to have a higher proportion of aberrant cells (mean of 65%) than benign (mean 61%) and hyperplasias (mean 53%) (Fig 2).

#### Regions with gains and losses

**Fig 3:**
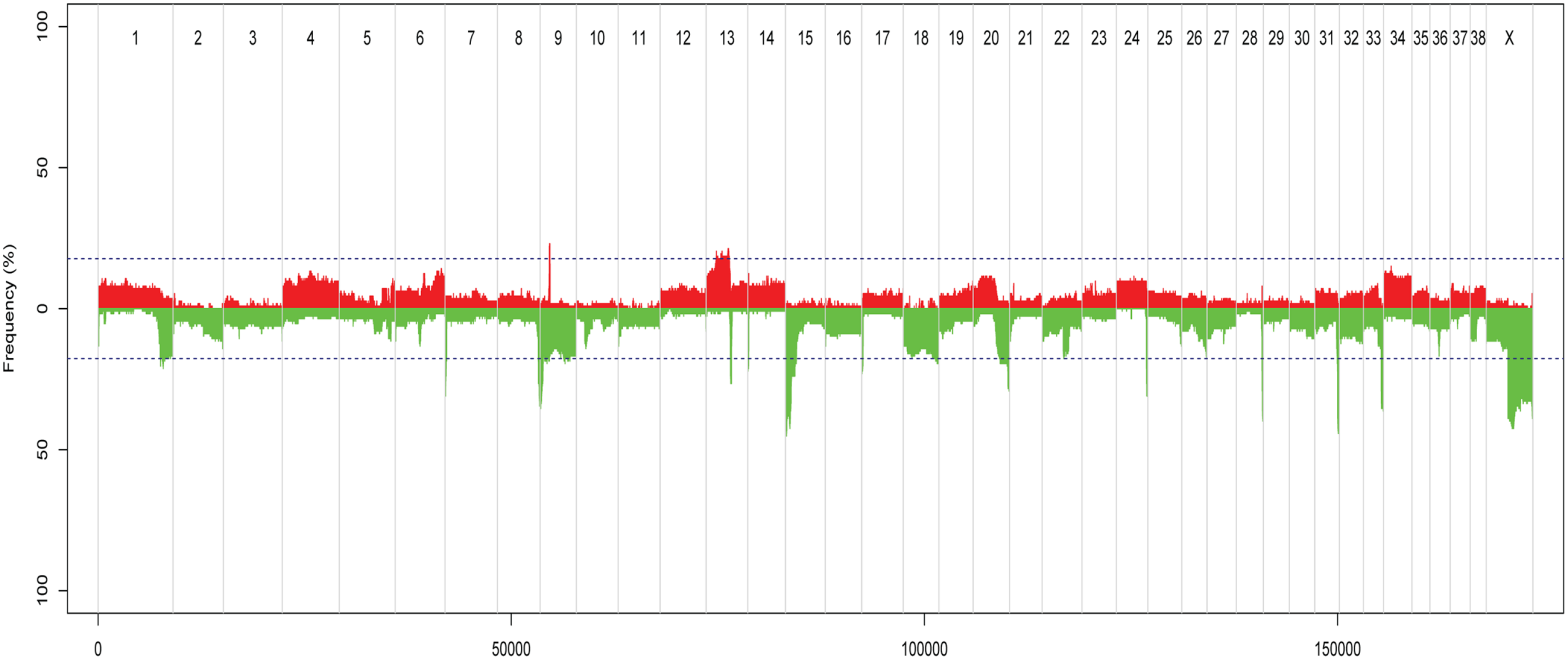
Recurring gains and losses across all tumour samples, according to ASCAT analysis. The figure shows recurring gains and losses across all tumour samples, relative to the ASCAT-estimate of ploidy for each tumour. Red = gains, green = losses, chromosome number along the x-axis, and frequency of tumours with aberration on the y-axis. Peaks outside the dotted blue lines are aberrations found in 20 or more samples.

According to the ASCAT analysis, losses were more highly recurrent than gains (recurrent region defined as aberration found in ≥ 20 tumours) (Fig 3). The two regions with the most frequent DNA copy number gains were located on CFA9 and 13. These were 1.4Mb and 19.4Mb in size, respectively. The well-known oncogene *MYC* was located in the most highly recurrently gained region on CFA13. No annotated canine gene was located in the very frequently gained region on CFA9 (19.863–20.922Mb). The region showed orthology to human chromosome (HSA) 17q (68.433–69.684Mb, hg19), that contained no annotated protein-coding genes in humans. However, the human non-protein coding gene *CASC17* (“cancer susceptibility candidate 17”) was located to this region. The regions that most often harboured deletions were located on CFA1, 7, 8, 9, 13, 14, 15, 17, 18, 20, 22, 24, 26, 28, 31, 33 and X. Nine of these were losses of chromosome ends/included telomeric regions (CFA8, 18, 20, 24, 26, 28, 31, 33 and X). The proportion of tumours with loss of the CFA18 chromosome end increased with malignancy. Such a loss occurred in approximately 8%, 13% and 30% of hyperplasias, benign and malignant tumours, respectively. For the rest of the chromosomes with loss of ends, these were also frequently seen in hyperplasias and benign tumours. One known cancer-associated gene, the tumour suppressor gene *MEN1*, was located in the highly recurrently lost regions. Losses in chromosome regions of well-known tumour suppressor genes like *BRCA1*, *BRCA2*, *BRIP1*, *CDH1*, *CHEK2*, *PTEN*, *RB1* and *TP53* were found in 18 (16.9%), 3 (2.7%), 16 (14.2%), 12 (10.6%), 10 (8.8%), 17 (15.0%), 11 (9.7%) and 4 (3.5%) tumours, respectively.

**Fig 4:**
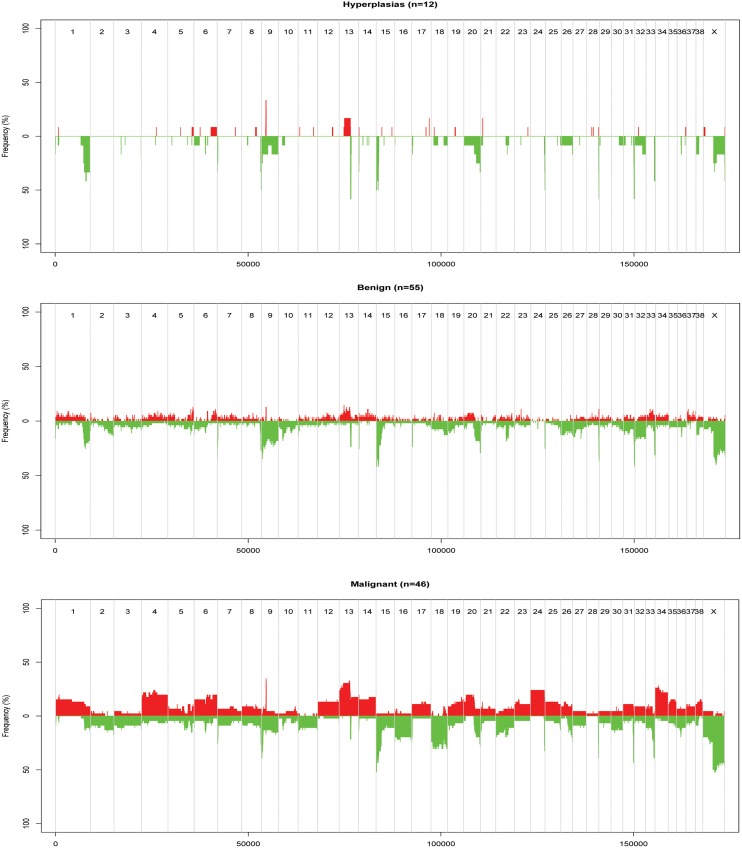
Recurring gains and losses in three subgroups of CMTs, according to ASCAT analysis. The figure shows recurring gains and losses in three subgroups of CMTs, relative to the ASCAT-estimate of ploidy for each tumour. Red = gains, green = losses, chromosome number along the x-axis, and frequency of tumours with aberration on the y-axis.

Similar to the PCF analysis, an increased frequency of aberrations was generally observed for increased malignancy of diagnosis for the ASCAT results (Fig 4). Gain of *MYC* was seen in two (16.7%) hyperplasias, 5 (9.1%) benign tumours and 14 (30.4%) malignant tumours. The number and frequency of aberrations also increased from lower grade malignant tumours to the most high-grade malignant tumours (data not shown). The most malignant tumours, solid and anaplastic carcinomas (n = 4), were found to have a higher frequency of whole-chromosome gains and losses compared to the others (data not shown).

### Significance analysis of aberrant regions

As described in the materials and methods, this analysis applying the method for GISTIC was performed for three different sample selections: in the first two data sets, the tumour pairs were split between selection one and two, while the single tumours were included in both selections. The analysis was also done for a third dataset including all tumours (all paired and single tumours). Significant amplification peaks were identified on CFA13 for all the three tumour sets. These significant regions included *MYC* for the “all tumours” group and for one of the selections with split tumour pairs. Significant regions of loss were observed for all three data sets on CFA13, 24, 28 and 31. For selection one, loss on CFA8 and for selection two, loss on CFA9 and CFA33, was also significant. However, the CFA33 loss was not significant considering all tumours together. These lost regions harbored genes like *COL9A3* (CFA24), *INPP5A* (CFA28), *CYP2E1* (CFA28), and *CRYAA* (CFA31).

### LOH and cnLOH

**Fig 5:**
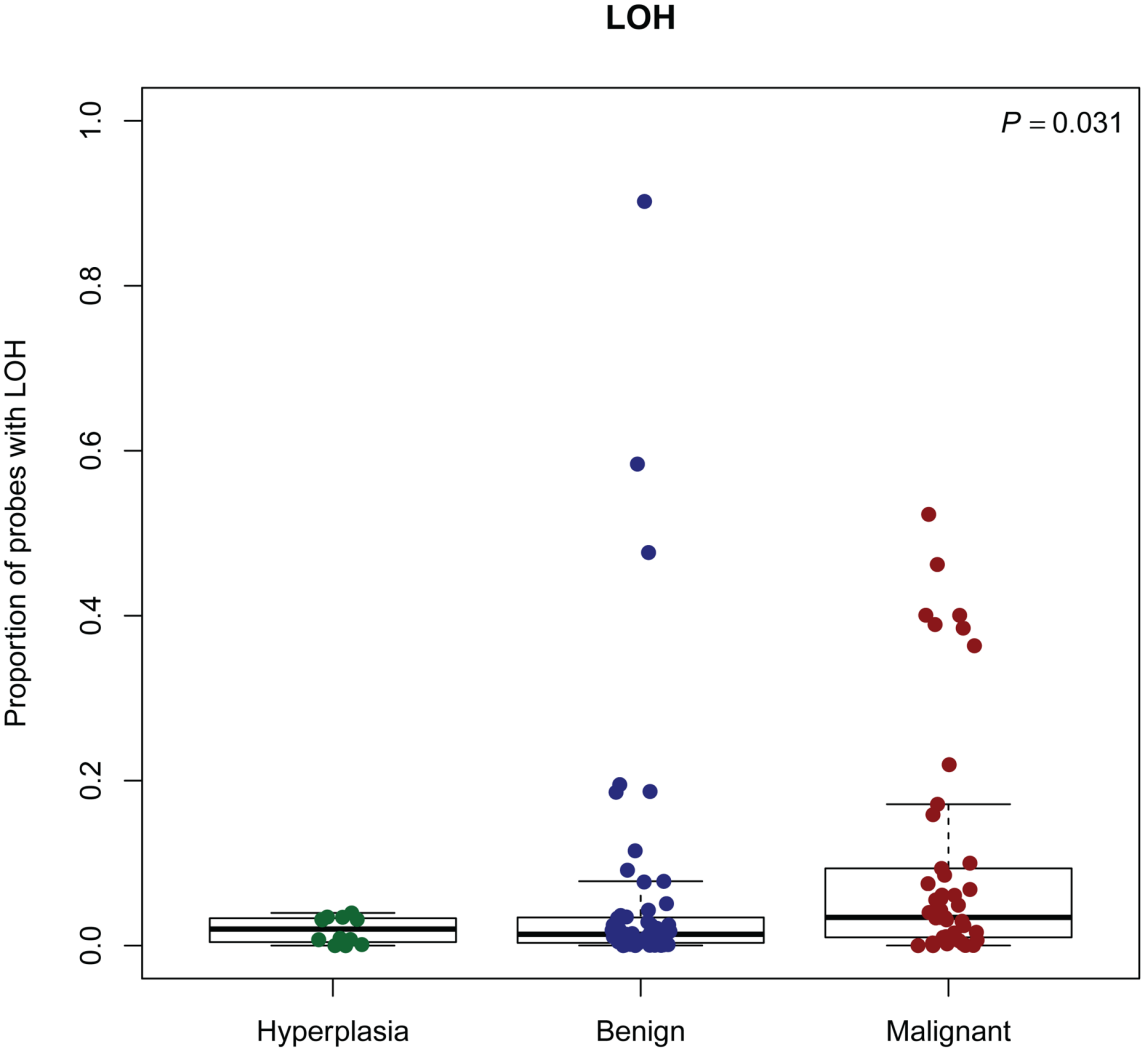
Proportion of probes with LOH in subgroups of CMTs, according to ASCAT analysis. LOH: Loss of heterozygosity. P: P-value from Kruskal-Wallis test.

**Fig 6:**
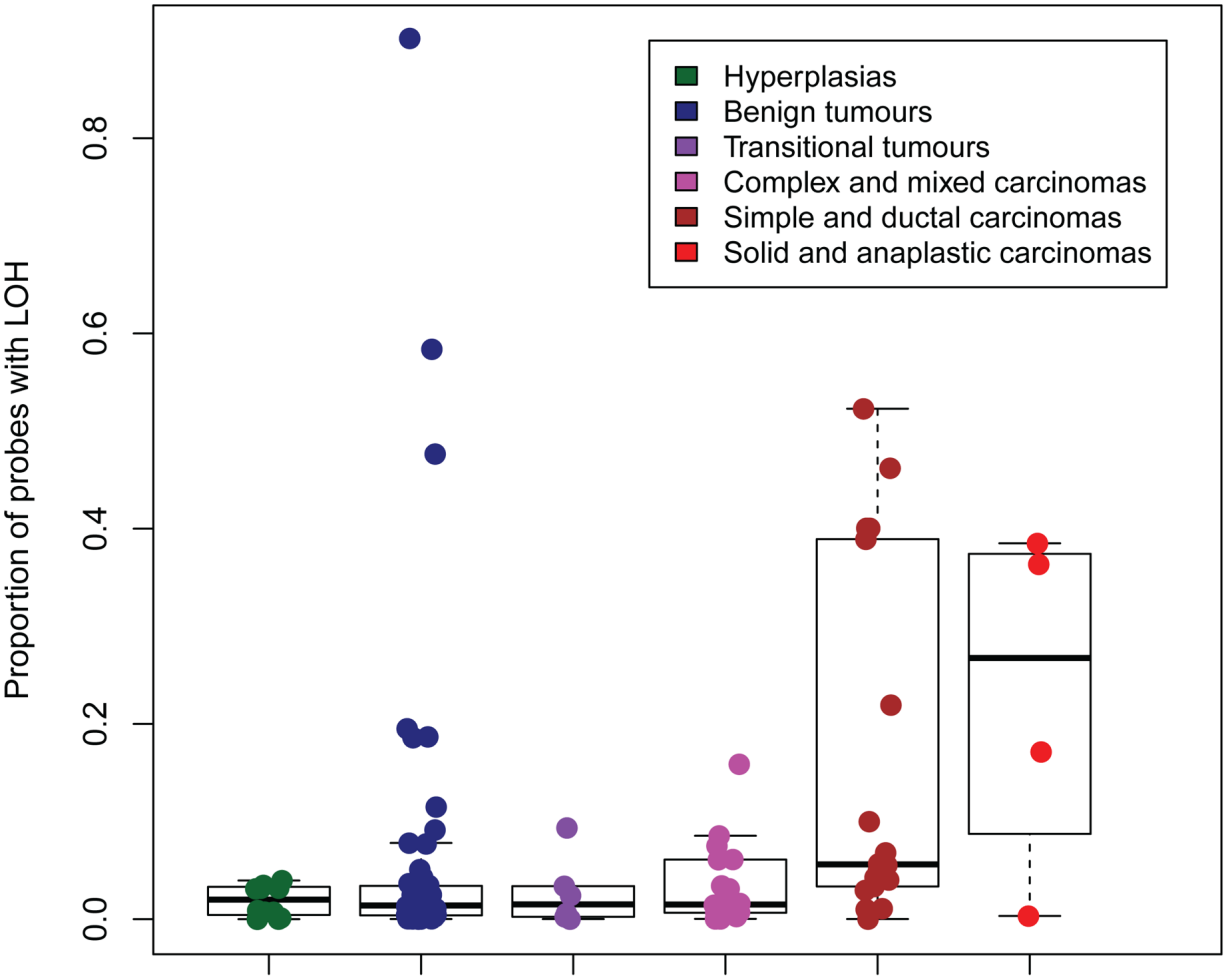
Proportion of probes with LOH in groups of morphologically similar tumours, according to ASCAT analysis. LOH: Loss of heterozygosity.

The regions with the highest frequency of loss of heterozygosity (LOH) were found on CFA8, 9, 13, 15, 18, 22, 24, 28, 31, 33, and X. These regions were orthologous to human chromosomes 14q32, 17q25, 8q24, 1p34/1p35, 7q21, 13q31, 20q13, 10q26, 21q22, 3q29 respectively [46]. A corresponding location in the human genome was not found for the region on CFAX. Analysis of the ASCAT output indicated that increased malignancy correlated with increased proportion of probes with loss of heterozygosity. This was observed for both the three groups of hyperplasias, benign and malignant tumours and the six groups of morphologically similar tumours. The two groups of most malignant tumours (including single, ductal, solid and anaplastic carcinomas) seemed to stand out from the rest (Figs 5 and 6). The same tendency was seen for copy-number neutral events (cnLOH) (data not shown). cnLOH was defined as an allelic bias for a SNP that was heterozygous in the germline, but without a change in total copy number from the tumour ploidy [24]. Several homozygous deletions were identified in the recurrently lost regions (Table 4). The genes *COL9A3*, *INPP5A* and *CYP2E1* were found in the regions with most frequently occurring homozygous deletions in each of the groups of hyperplasias, benign and malignant tumours. *PTEN* was the only gene from the Cancer Gene Census List [44] that was found in regions with homozygous deletions in at least five tumours. Homozygous deletion of *PTEN* was found in five tumours, four of them malignant. These four were one anaplastic carcinoma, one simple tubular carcinoma and two complex carcinomas.

**Table 4:** Genes in regions of homozygous deletions occurring in at least five tumours according to ASCAT.

Gene	Human chromosome	Canine chromosome	Number of tumours with homozygous deletion
*TNFRSF12A*	16	6	5
*PKD1*	16	6	6
*SEPX1*	16	6	6
*LOC448801*	-	6	6
*LOC100049001*	-	6	6
*HAGHL*	16	6	6
*AMN*	14	8	8
*PDE6G*	17	9	7
*ACTG1*	17	9	7
*SGSH*	17	9	6
*TIMP2*	17	9	6
*ELANE*	19	20	5
*MADCAM1*	19	20	5
*COL9A3*	20	24	17
PTEN*	10	26	5
*LIPF*	10	26	5
*INPP5A*	10	28	15
*CYP2E1*	10	28	15
*TFF3*	21	31	5
*TFF2*	21	31	5
*TFF1*	21	31	5
*CRYAA*	21	31	11

* Gene found in the Cancer Gene Census list [40].

### Histopathological parameters

**Fig 7:**
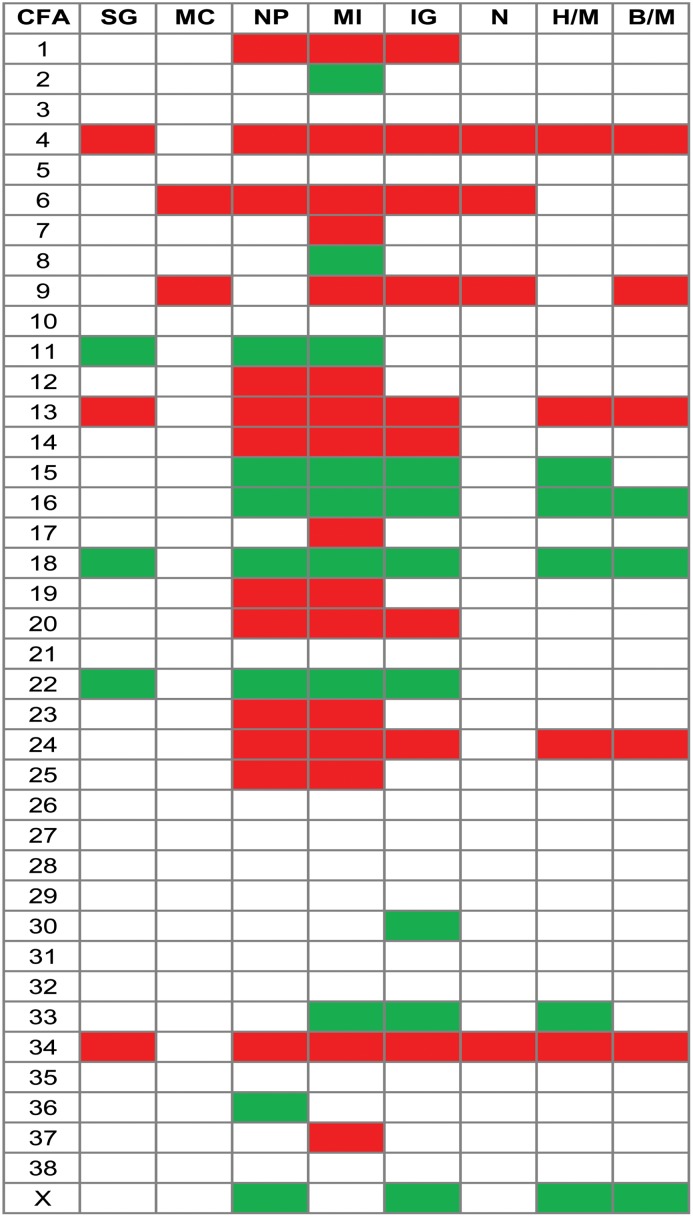
Chromosome-wise gains (red) and losses (green) for different histopathological parameters. Gain/loss frequencies from the ASCAT analysis were used for these calculations. Only chromosomes with regions with a CNA-frequency difference ≥20% are shown (see Materials and Methods). The corresponding contrasts between hyperplasias/malignant tumours and benign/malignant tumours are shown for comparison. There were no regions with difference in frequency of loss ≥20% for compared categories of the parameters myoepithelial cells and necrosis. CFA: canine chromosome. SG: Solid growth (no/yes), MC: Myoepithelial cells (yes/no), NP: Nuclear pleomorphism (no/moderate-severe), MI: Mitotic index (0/above 9 pr HBF), IG: Invasive growth, tumour stroma (no/yes), N: Necrosis (no/yes), H/M: Hyperplasia vs malignant, B/M: Benign vs malignant.

To further dissect the CNAs with possible involvement in CMT pathogenesis, we looked at chromosomal gains and losses associated with some histopathological parameters that are used for evaluating tumour progression/malignancy. In general, the frequency of aberrations increased if solid growth, invasive growth in tumour stroma and necrosis was present in the tumour. Tumours with a myoepithelial cell component had a lower occurrence of aberrations than tumours without such cells. The occurrence of CNAs also increased in tumours with a moderate to severe nuclear pleomorphism compared to none and with a mitotic index above nine per ten HPF compared to zero. Fig 7 delineates the chromosome-wise CNA pattern for various morphological traits and shows that some aberrations might be associated with specific histopathological parameters. Only chromosomes with regions with an aberration frequency difference between parameter subgroups of ≥20% (see Materials and Methods) are shown in Fig 7. Many of the same chromosomes were affected by gains/losses for different histopathological parameters. However, gains in regions on CFA7, 17 and 37 and loss of regions on CFA2 and 8 seemed more specific to an increase in mitotic index. An increased frequency of losses in regions on CFA36 was seen for moderate to severe nuclear pleomorphism compared to none, while loss of regions on CFA30 was associated with invasive growth in tumour stroma.

The chromosome region aberrations associated with changes in the histopathological parameters were studied for well-known cancer-associated genes. *MYC* was located in amplified regions of CFA13 associated with moderate to severe nuclear pleomorphism, high mitotic index and invasive growth into tumour stroma. The gained region on CFA20 that was associated with moderate to severe nuclear pleomorphism contained *FHIT*. *RB1* was located in the frequently lost region on CFA22 associated with solid growth, moderate to severe nuclear pleomorphism, high mitotic index and invasive growth into tumour stroma. The tumour suppressor gene *SDHB* was located in the region on CFA2 that was associated with high mitotic index. The lost region on CFA18 was associated with solid growth and invasive growth into tumour stroma included the *MEN1* gene.

## Discussion

In this study we have identified a large number of recurrent aberrations. This is in line with previous studies of CNAs in human cancers [24,26,27]. There are few reports on CNAs in CMTs, however, both a recent study of genomic aberrations in canine carcinomas as well as cytogenetic analysis of cell culture originating from CMTs showed widespread genomic alterations [34,54]. Array-comparative genomic hybridization (aCGH) studies of canine osteosarcomas and canine histiocytic sarcomas have also revealed highly complex tumours, with aberrations ranging from numerous single locus CNAs to losses or gains of entire chromosomes [55,56]. The wide range in number and types of chromosome level alterations in CMTs is expected and probably reflects a combination of selection and particular failures in genome surveillance mechanisms. The observation that the frequency of aberrations seemed to be positively correlated with increased histopathological malignancy in all the three subgroupings of the material is interesting. However, the tumour number in some of the carcinoma groups is low, and for these the results must be interpreted with caution. Recurrent CNA losses were more frequent than gains in the mammary tumours in this study, something which was also previously seen in canine histiocytic sarcomas [55] and two of the single canine mammary tumours studied by Bech et al [34]. Also, the observations of a high number of copy number gains on CFA13 and few aberrations on CFA23 are similar to what has been found in other canine tumour types [56,57]. CFA16 was most often affected by recurrent copy number losses in canine osteosarcoma and histiocytic sarcoma [55,56], while the proximal end of CFA27 was reported to be deleted in four out of five canine mammary carcinomas [34]. However, other chromosomes were affected by more deletions in the CMTs of the present study. This might reflect differences in genetic contributors between different tumours and cancer types, as has been shown in recent studies of signatures of mutational processes in human cancers. These identified both distinct variations and similarities regarding classes of mutations and mutational patterns between (and within) different tumour types [58,59].

In addition to CNAs, aneuploidy clearly distinguishes cancer cells from normal cells. The proportions of aneuploid hyperplasias and benign tumours in the present study were in line with previous reports based on flow cytometric analysis of CMTs, while the proportion of aneuploid malignant tumours in our study was lower. Rutteman et al. observed aneuploidy in 68.1% of mammary cancers and 17.4% of nonmalignant tumours [60], while the corresponding numbers in a study Hellmén et al. were 54% of the histologically malignant, 13% of the histologically benign tumours and 22% of the dysplastic mammary glands [61]. Aneuploidy was reported in 45% of early-stage human breast carcinomas, which is somewhat higher than in the malignant mammary tumours in our study [24]. The differences between the studies regarding ploidy of malignant tumours might be due to biological variation, differences in analysis methods, change of classification guidelines of CMTs over time and different pathologists evaluating the tumours. Although not large, we found a tendency of increased aneuploidy with increased malignancy. Aneuploidy in benign tumours might reflect a malignant potential in these tumours that is not yet shown by the histopathomorphology [60]. While aneuploidy has been negatively associated with survival rates of dogs with diagnosed malignant mammary tumours [62], others found no relationship between the incidence of aneuploidy and histological tumour type, histological malignancy grade, nuclear grade or steroid receptor presence [60]. We observed only two mammary tumours with hypodiploidy, in contrast to both Rutteman et al. and Cornelisse et al. who reported hypodiploidy to be more frequent in dogs than in humans as measured by flow cytometry [60,63]. It has been hypothesized that hypodiploidy might be an early event in the canine mammary tumourigenesis, followed by multidiploidy or polyploidy during tumour progression [60,62]. This is partly supported by studies of human breast cancer: e.g. the basal-like subtype often has a ploidy around 3n, and it has been suggested that the genomes of these tumours initially are reduced from diploid to a partial haploid state (around 1.5n) before a whole-genome duplication to a ploidy around 3n [24]. A similar explanation might apply to malignant tumours in our study with a ploidy ~3. Studies of human cancers have also suggested that aneuploidy develops through an unstable tetraploid intermediate and that genome doubling could act as a precursor to chromosomal instability [64]. The tendency of an increased proportion of tetraploidy in benign CMTs compared to malignant and hyperplasias fits well with this theory.

The identified cancer-associated genes in the recurrently gained regions in the present survey were of dominant function/oncogenes only. Since copy number alterations in human breast cancer have been shown to directly cause global deregulation of gene expression [65], and thereby may contribute to the development or progression of cancer, oncogenes can be expected to be located in amplified chromosomal regions of the tumour and genes with recessive molecular function/tumour suppressor genes in deleted regions. Similarly to surveys of canine mammary carcinomas and canine osteosarcomas [34,56], the frequently gained region on CFA13 included the *MYC* gene in the mammary tumours of the present study. The *MYC* region amplification was also among the identified significant aberrations. The proto-oncogene *MYC* encodes a nuclear transcription factor (phosphoprotein) that plays an integral role in a variety of cellular processes, such as cell growth, proliferation, metabolism, differentiation and apoptosis [66]. *MYC*, located on chromosome 8q24 in humans, harbors multiple, upstream risk loci for human prostate, bladder, breast, and colorectal cancer [67]. In human breast cancer, amplification of the *MYC* gene is a quite frequent event [68,69]. Amplification of *MYC* seems to occur relatively late in tumourigenesis and is consistently observed in aggressive forms of disease, correlating with poor prognosis and distant metastasis [68]. *MYC* amplification has also been suggested to be a driver of and selected for in the metastatic process [68]. The fact that we observed a higher proportion of the malignant than benign mammary tumours with recurrent amplification in the region of *MYC* correlates well with the known involvement of *MYC* in human breast cancer. *PTEN* was located in lost regions in mammary tumours of the present study, which is in line with previous studies of canine cancers [34,55,56]. Our observation of homozygous deletions of *PTEN* in several malignant mammary tumours further indicates loss of *PTEN* as an important event in CMT development. *PTEN* is a tumour suppressor gene that encodes the protein phosphatidylinositol-3,4,5-trisphosphate 3-phosphatase and plays an important role in inducing cell cycle arrest, programming apoptosis, regulation of cell adhesion, migration, and differentiation [70]. The *PTEN* gene is also one of the most commonly mutated genes in human cancer [70], among them breast cancer [27,71], and often suffers loss of heterozygosity (LOH) [72]. In addition to the amplification and deletion of specific genes, CNAs might as well disrupt critical stoichiometric relationships in cell metabolism and physiology (e.g. proteasome, mitotic spindle), possibly promoting further instability and directly contributing to tumour development or progression [65]. The detected copy number aberrations in regions without known annotated genes in the CMTs of this study, e.g. the region on CFA9, could have such disruptive effects, contain hitherto unknown genes/sequences associated with tumourigenesis or be random events due to genomic instability.

The regions with the highest occurrence of LOH in our study did not show orthology to any of the chromosome arms with frequent LOH found in in human breast cancer (HSA8p, 11q, 16q, 17p) [24]. However, similar to human breast cancer [24], many of the genomic regions with a higher frequency of loss also harbored copy-number neutral events in the CMTs. The genes *COL9A3*, *INPP5A* and *CYP2E1*, located in chromosomal regions for significant aberrations and the highest frequency of homozygous deletions in our study, were not in the Gene Cancer Census list [44]. Still, previous reports on cancer-association in humans and the function of the encoded protein, at least for *INPP5A* and *CYP2E1*, propose that they may play a part in the CMT pathogenesis. *COL9A3* encodes one of the three alpha chains of type IX collagen, the major collagen component of hyaline cartilage. A homozygous missense mutation in this gene has been detected in a human breast carcinoma [73,74]. *INPP5A* encodes membrane-associated type I inositol 1,4,5-trisphosphate (InsP3) 5-phosphatase which is involved in mediating cell responses to various stimuli [73]. Mutations in *INPP5A* have been found in several cancers [74], and loss of the gene has been identified as an early event of human cutaneous squamous cell carcinoma [75]. This is possibly also the case for CMTs, as we found the gene to be homozygously deleted in hyperplasias, benign and malignant tumours. *CYP2E1* encodes a member of the cytochrome P450 superfamily of enzymes that is involved in metabolism of both endogenous and exogenous substrates. Polymorphisms in the *CYP* genes are associated with risk of several cancer types [76]. *CYP2E1* expression has been found to be both significantly lower in malignant breast tissue compared to normal tissue in humans [77] and also lower in tumours of clinical stage I compared to stage II-IV [78]. The loss of *CYP2E1* in CMTs is in line with these findings in human breast cancer.

As expected, some of the aberrations in regions of oncogenes and tumour suppressor genes were common for changes in subgroups of histopathological parameters and tumour diagnosis groups. In addition, loss of the well-known tumour suppressor *RB1* was found to occur at a higher frequency in tumours that had developed characteristics such as solid growth, moderate to severe nuclear pleomorphism, high mitotic index and invasive growth into tumour stroma. Aberrations in this gene is known from a variety of human tumours [79]. Highly recurrent loss of *RB1* was also found in canine histiocytic sarcomas [55]. The frequency of loss of this gene, located on CFA22, was not as prominently different between the hyperplasias, benign and malignant tumours as for the histiopathological parameter subgroups. This fact illustrates how studies of subgroups of histopathological parameters in more detail, in addition to histopathological diagnosis, have the potential to elucidate aberrations/genes associated with tumour progression.

The main copy number aberration results of the present survey were confirmed by all three algorithms used to study the CMTs, increasing the credibility of the findings. Precautions to reduce bias and technical artifacts through GC correction and normalization of the data prior to analysis, combined with the limits for aberration calls in the algorithms, and finally focusing on only the most highly recurrent aberrations in this large material of tumours, greatly reduce the chance of reporting false aberration results or random findings. The observation of similar aberrant regions and genes as previously described in both humans and canine cancers, especially the recent CMT survey by Beck et al. [34], also supports the validity of our results. However, functional evaluation of the copy number aberrations of the present study would greatly add to our understanding of their importance in CMT development and should be addressed in future studies.

## Conclusion

The present study found that CNAs are common in CMTs and that the frequency of aberrations and LOH increases with histopatholgical malignancy of the tumours. Amplification of *MYC* and loss of *PTEN*, possibly also loss of *COL9A3*, *INPP5A*, *CYP2E1*, and *RB1*, may be important events in CMT development and progression. The recurrent amplification on CFA9 should be more extensively analyzed for its implication for CMT pathogenesis, and analysis of chromosomal aberrations associated with histopathological parameters in more detail may aid in identifying specific genes associated with CMT progression. Several similarities between human and canine mammary neoplasias in case of recurrent CNAs further support the dog as a valuable model for this disease in humans.

## Supporting Information

S1 Table Genes identified by PCF in recurrently gained regions.pdf. All genes identified by PCF in the regions of gains found in ≥20% of the tumour samples.
(PDF)

S2 Table Genes identified by PCF in recurrently lost regions. All genes identified by PCF in the regions of loss found in ≥20% of the tumour samples.
(PDF)
