# Supplementary material for: Canine Mammary Tumours Are Affected by Frequent Copy Number Aberrations, including Amplification of MYC and Loss of PTEN
Source: PLoS One. 2015 May 8;10(5):e0126371. doi: 10.1371/journal.pone.0126371 (PMC4425491; doi:10.1371/journal.pone.0126371)
Supplement: S1 Table — All genes identified by PCF in the regions of gains found in ≥20% of the tumour samples. (PDF) [file pone.0126371.s001.pdf]

Supplementary file 2. Genes identified by PCF in recurrently lost regions.

All genes identified by PCF in the regions of loss found in ≥20% of the tumour samples.

Gene ID	Transcript ID	CFA	Position
ATP4A	NM_001003342	1	120013195-120025391
ELSPBP1	NM_001002931	1	111045499-111073002
LHB	NM_001197033	1	110270318-110271493
RAB4B	NM_001003275	1	115911880-115920510
TYROBP	NM_001197117	1	119666321-119669486
KRTDAP	NM_001024642	1	120075785-120078977
SLC7A9	NM_001048109	1	122118197-122141419
CEACAM23	NM_001097552	1	115366831-115374164
KLK9	NM_001171773	1	108747688-108752689
CAECAM1	NM_001113459	1	114661581-114672522
CEACAM28	NM_001097546	1	114704752-114726772
FTL	NM_001024636	1	110306078-110307172
APOC1	NM_001197048	1	113410464-113412850
CRX	NM_001003049	1	111135799-111146901
DHDH	NM_001003160	1	110321816-110332522
TGFB1	NM_001003309	1	115538140-115552350
KLK1	NM_001003262	1	108891048-108910255
CYP2A7	NM_001048027	1	115809009-115815247
C5AR1	NM_001003373	1	111545381-111561146
KLK14	NM_001165879	1	108691776-108696178
FXYD1	NM_001195143	1	120344684-120349031
NTF4	NM_001190429	1	110258667-110264451
KLK4	NM_001197169	1	108833501-108836975
KLK5	NM_001197167	1	108796270-108804096
KLK6	NM_001197166	1	108781688-108789385
APOC2	NM_001003368	1	113395947-113396358
CYP2B6	NM_001006652	1	115726060-115741796
KLK2	NM_001003284	1	108863121-108866361
SCN1B	NM_001025398	1	120412876-120419113
HSD17B14	NM_001047981	1	110405474-110422937
CEACAM1	NM_001097557	1	114794550-114809492
CEACAM24	NM_001097554	1	114637512-114648111
CEACAM30	NM_001097553	1	114704757-114726760
CYP2A13	NM_001037345	1	115840747-115846750
AKT2	NM_001012340	1	116297439-116315357
HAMP	NM_001007140	1	120214047-120215104

Gene ID	Transcript ID	CFA	Position
VN1R4	NM_001013852	1	106636393-106637344
FOSB	NM_001013844	1	112979948-112984081
KLK8	NM_001197162	1	108754819-108760279
VASP	NM_001003256	1	112941416-112954090
SEPW1	NM_001115012	1	111171331-111175440
ZNF331	NM_001003331	1	106592495-106606489
FLT3LG	NM_001003350	1	109902853-109912340
TNNI3	NM_001003041	1	105551207-105554381
KLK10	NM_001197158	1	108740501-108743780
KLK7	NM_001197165	1	108771219-108774343
RPL18	NM_001251958	1	110575572-110579311
SNRPD2	NM_001252412	1	112782530-112788031
RABAC1	NM_001003014	1	115251508-115254103
RHPN2	NM_001003008	1	121974059-122031890
ARHGAP35	NM_001003022	1	111838427-111919920
BAX	NM_001003011	1	110312434-110313918
FUCA1	NM_001003250	2	78558119-78569734
TMEM57	NM_001038648	2	77289114-77349517
SMN	NM_001003226	2	57469028-57509747
EPB41	NM_001003362	2	74366507-74475019
ANKHD1	NM_001204095	2	38468098-38586987
TAS1R2	NM_001031819	2	82613795-82632671
CASP9	NM_001031633	2	84932265-84951816
OCLN	NM_001003195	2	57373330-57418462
HTR1D	NM_001003280	2	79090802-79092465
DDOST	NM_001003321	2	81162313-81169563
CDC42	NM_001003254	2	80045251-80061916
RHCE	NM_001048036	2	77358318-77394841
CCL17	NM_001003051	2	61881387-61883900
CD52	NM_001003240	2	76590942-76592562
EIF4EBP3	NM_001202524	2	38592552-38594490
SLC23A1	NM_001109957	2	37548456-37556643
PADI6	NM_001097547	2	83785360-83804180
PDPN	NM_001003220	2	86451127-86479288
APITD1	NM_001204929	2	88363460-88372550
HMGN2	NM_001003101	2	76470557-76474302
SNHG3	NR_038068	2	74863974-74866717
RCC1	NM_001205212	2	74838917-74855106
SDHB	NM_001252217	2	84054658-84086379
BTF3	NM_001252133	2	59211026-59214795

Gene ID	Transcript ID	CFA	Position
ALPL	NM_001197137	2	80453595-80468819
RPL11	NM_001253907	2	78673771-78678176
PFDN1	NM_001252417	2	38304187-38369479
ID3	NM_001003025	2	78773211-78774339
HGFAC	NM_001031815	3	63726781-63735023
TJP1	NM_001003140	3	41851670-41935267
CKMT2	NM_001145214	3	28894033-28909762
F2RL1	NM_001145991	3	32688583-32697471
TMED11	NM_001003286	3	94267667-94284327
VIMP	NM_001114757	3	42654233-42663177
AP3B1	NM_001002974	3	31369512-31635611
ARSB	NM_001048133	3	30752753-30917513
PDE6B	NM_001002934	3	94573131-94601412
PLAU	NM_001194952	4	27539754-27544509
MYOZ1	NM_001048095	4	27369426-27374791
TACR2	NM_001012617	4	23724516-23736838
BMPR1A	NM_001145150	4	37709857-37770882
LMAN2	NM_001003258	4	39194253-39214922
PRF1	NM_001197182	4	24707270-24709818
CYBA	NM_001100290	5	67689862-67698054
TP53	NM_001003210	5	35557005-35560761
TNFRSF18	NM_001190744	5	59396105-59398658
TNFSF13	NM_001205169	5	35478482-35480550
TNFSF12	NM_001205168	5	35469355-35476881
ZBTB4	NM_001205162	5	35380261-35387089
HSF4	NM_001048121	5	85204147-85208679
TAS1R3	NM_001031821	5	59504715-59507879
CA6	NM_001002999	5	65357101-65378480
SREBF1	NM_001197083	5	44662652-44685553
MYH3	NM_001113712	5	37908501-37930194
B3GALT6	NM_001048137	5	59422879-59424313
GP1BA	NM_001003083	5	34678673-34679655
LEPR	NM_001024634	5	47690110-47755068
GALNS	NM_001048120	5	67531292-67559268
SLC2A4	NM_001159327	5	35234684-35240612
GUCY2D	NM_001003207	5	35838279-35853510
ALDH3A1	NM_001082420	5	43466509-43474545
SLC12A4	NM_001010952	5	84541294-84563234
MYH2	NM_001076795	5	37804508-37829610
FLCN	NM_001127611	5	45177319-45196434

Gene ID	Transcript ID	CFA	Position
IL12RB2	NM_001025399	5	46199180-46262056
SLC1A7	NM_001197159	5	58784126-58826756
MYH8	NM_001077235	5	37690077-37717158
MYH4	NM_001076794	5	37737037-37760660
FAM151A	NM_001167659	5	57502628-57513435
LOC479649	NM_001197055	5	78509247-78512666
SCAMPER	NM_001003197	5	64430752-64431993
MYH1	NM_001113717	5	37775053-37799628
DIO1	NM_001007126	5	58083735-58100402
MC1R	NM_001014282	5	66692397-66693344
RCVRN	NM_001014281	5	37317091-37324175
TEKT1	NM_001003097	5	33594352-33607659
NPHP4	NM_001135788	5	62819749-62935638
ADORA2B	NM_001002944	5	42885002-42908791
PLA2G15	NM_001002940	5	84277733-84288514
KCNJ12	NM_001242714	5	45850780-45861308
EIF4A1	NM_001251942	5	35491364-35496414
CMTM1	NM_001252345	5	85704652-85716970
CKLF	NM_001252340	5	85717448-85734365
SSU72	NM_001252325	5	59621760-59651089
RPL26	NM_001252400	5	36146420-36152544
GNB1	NM_001003236	5	59780353-59818298
GFI1	NM_001012719	6	59531051-59540188
HAGHL	NM_001204219	6	42895096-42897442
TNFRSF12A	NM_001193299	6	41208321-41209735
PKD1	NM_001006650	6	41879345-41906885
DNASE1	NM_001002946	6	40638694-40641442
PAM16	NM_001003391	6	40082453-40089474
LOC448801	NM_001005260	6	42518906-42520868
SEPX1	NM_001114749	6	42022812-42027551
GUSB	NM_001003191	6	3732316-3745868
LOC100049001	NM_001097555	6	42546599-42548024
ARHGEF2	NM_001002995	7	44806269-44821436
KRTCAP2	NM_001160122	7	45328170-45330529
S100A4	NM_001003161	7	46471121-46473134
CYB5R1	NM_001160425	7	3230142-3232672
CHI3L1	NM_001177807	7	3026210-3032819
ADORA1	NM_001003279	7	3044727-3073015
MUC1	NM_001194977	7	45313308-45317624
SSR2	NM_001003269	7	44523597-44772382

Gene ID	Transcript ID	CFA	Position
TPM3	NM_001252211	7	46019881-46042029
RPS27	NM_001252286	7	46124148-46125342
PKLR	NM_001256018	7	45237329-45244826
RHBG	NM_001003017	7	44506766-44517035
TNNT2	NM_001003012	7	4517491-4523990
DIO3	NM_001164188	8	72739426-72741528
AMN	NM_001002960	8	73515692-73850599
LOC403829	NM_001003189	9	52953168-52956967
GNG10	NM_001205002	9	50610452-50611556
CCL7	NM_001010960	9	42316308-42317774
FUT7	NM_001005379	9	51940728-51941796
GBGT1	NM_001003193	9	54616142-54625249
GRIN1	NM_001008717	9	51823758-51847073
PTGES2	NM_001131050	9	58687501-58693036
SLC2A8	NM_001131048	9	59266043-59273162
SOCS3	NM_001031631	9	5856588-5857266
ACTG1	NM_001003349	9	3625258-3627598
DBH	NM_001005263	9	53342443-53361759
CCL13	NM_001003966	9	42260699-42261987
PDE6G	NM_001003235	9	3514785-3517598
TIMP2	NM_001003082	9	5519215-5531215
ST6GALNAC2	NM_001097559	9	7152731-7163101
CCL8	NM_001005255	9	42270939-42272458
AK1	NM_001131047	9	58887591-58892542
LCN1	NM_001003190	9	53042726-53045221
UNK	NM_001003390	9	7765532-7799023
HN1	NM_001099943	9	8319187-8337203
SLC6A4	NM_001110771	9	47549262-47572016
SGSH	NM_001003114	9	4530025-4536122
ZACN	NM_001010955	9	7553268-7556569
SPACA3	NM_001197087	9	43417625-43425242
TBX2	NM_001005249	9	38559524-38567775
GGTA1	NM_001206937	9	64358679-64387362
CRYBA1	NM_001080899	9	46697895-46703940
CCL3	NM_001005251	9	41014114-41015541
CCL4	NM_001005250	9	41001745-41003095
BIRC5	NM_001003348	9	5927500-5932654
LGALS9	NM_001003345	9	45480186-45495876
SERPINF1	NM_001077588	9	49216446-49227785
CYGB	NM_001077587	9	7189310-7198202

Gene ID	Transcript ID	CFA	Position
RHBDF2	NM_001077438	9	7224401-7235821
AANAT	NM_001077437	9	7237321-7238745
SRP68	NM_001003271	9	7565597-7602199
PTGES	NM_001122854	9	57416128-57426889
PTGDS	NM_001003131	9	51980425-51983700
PRCD	NM_001097560	9	7183512-7186824
OR1E2	NM_001014287	9	50644159-50645101
CCL1	NM_001005252	9	42255813-42258454
CCL2	NM_001003297	9	42329860-42331603
NOS2	NM_001003186	9	45497307-45527183
LHX3	NM_001197187	9	52581388-52586896
ST6GALNAC1	NM_001097556	9	7099096-7121911
DNM1	NM_001131049	9	58596880-58642323
ADAMTS13	NM_001242712	9	53176743-53204933
RPL12	NM_001251956	9	59239350-59243225
TRPV1	NM_001003970	9	50732902-50759074
SUMO2	NM_001252289	9	8295839-8308682
LOC480600	NM_001253735	9	41028964-41030613
SYNGR2	NM_001252581	9	5969744-5973959
POLDIP2	NM_001253903	9	45923139-45932924
PTGS1	NM_001003023	9	63536549-63558202
TUBD1	NM_001003024	9	37678226-37708890
COR1A3	NM_001256454	9	50447820-50448750
GALK1	NM_001003104	9	7816346-7822625
TBX4	NM_001003006	9	38487908-38513488
CCL5	NM_001003010	9	41137812-41144256
SELO	NM_001137603	10	20015345-20023245
CYB5R3	NM_001048084	10	25893768-25912155
CYP2D15	NM_001003333	10	26319100-26323222
PDGFB	NM_001003383	10	28865786-28884090
ARHGAP8	NM_001204405	10	24144401-24192394
SSTR3	NM_001025620	10	30401600-30402878
LGALS1	NM_001201488	10	30004664-30007894
PPARA	NM_001003093	10	22995235-23060884
MYH9	NM_001110767	10	31135176-31194505
ARSA	NM_001048083	10	19679653-19682073
PRR5	NM_001204407	10	24224301-24250417
MCHR1	NM_001003038	10	27592878-27595253
EIF3L	NM_001251940	10	29829959-29857229
RPL3	NM_001252159	10	28805937-28812336

**Gene ID**	**Transcript ID**	**CFA**	**Position**
ST13	NM_001252418	10	27445298-27477066
PROP1	NM_001020807	11	5743341-5745960
STOM	NM_001142670	11	77297769-77311639
CANX	NM_001003232	11	4969905-5001905
TMED7	NM_001204338	11	8346180-8360744
TICAM2	NM_001204337	11	8316298-8317015
BAK1	NM_001020808	12	5954905-5958144
MAPK14	NM_001003206	12	8146556-8219757
LTB	NM_001033510	12	4082529-4084325
DLA-64	NM_001014378	12	3986744-3990139
DDX39B	NM_001014377	12	4035769-4046166
DLA88	NM_001014767	12	3895615-3898863
DLA-DRA	NM_001011723	12	5137040-5140595
DLA-DMA	NM_001048099	12	5520361-5523922
PIM1	NM_001146177	12	9214019-9218449
PSMB9	NM_001048086	12	5435890-5441455
ATP6V1G2	NM_001014376	12	4050049-4051289
TNF	NM_001003244	12	4077732-4079597
PFDN6	NM_001048089	12	5732947-5734244
PSMB8	NM_001048085	12	5422841-5426218
DLA-DQA1	NM_001011726	12	5224566-5230986
RING1	NM_001048128	12	5674963-5678874
LY6G5C	NM_001003166	12	4162833-4166762
DLA-DQB1	NM_001014381	12	5248205-5254119
PPARD	NM_001048102	12	7567688-7580734
DLA-DOB	NM_001048127	12	5381511-5387118
B3GALT4	NM_001080722	12	5722705-5724383
HLA-DRB1	NM_001014768	12	5155325-5252520
TAPBP	NM_001048101	12	5743426-5755312
HMGA1	NM_001003387	12	6531341-6541048
CLPS	NM_001003287	12	7912138-7914208
SLC39A7	NM_001048100	12	5667316-5670913
NFKBIL1	NM_001014375	12	4052346-4060409
AGER	NM_001048081	12	4576954-4579803
DLA-12	NM_001014379	12	3936492-3939842
VPS52	NM_001048088	12	5700893-5716217
BRD2	NM_001048087	12	5537419-5549415
DLA-DMB	NM_001271070	12	5506710-5511766
CYP21A2	NM_001003335	12	4453446-4456470
HSP70	NM_001003067	12	4288013-4290315

Gene ID	Transcript ID	CFA	Position
RPS18	NM_001048082	12	5716343-5722043
FKBP1A	NM_001252190	12	7083390-7097866
CLIC1	NM_001252138	12	4213087-4218539
RPS10	NM_001252045	12	6716192-6722358
RPL10A	NM_001252145	12	7603071-7605453
NUDT3	NM_001252389	12	6569720-6689235
RPP21	NM_001252381	12	3331809-3333543
ARF1	NM_001251927	14	3786149-3787098
AKR1B1	NM_001252416	14	5942194-5957240
COR2T18	NM_001256452	14	5645405-5646368
PPT1	NM_001010944	15	5864001-5889887
RPPH1	NR_002313	15	20710054-20710753
APEX1	NM_001145119	15	20791700-20794315
SLC2A1	NM_001159326	15	3463778-3492831
RSPO1	NM_001130838	15	7872863-7885429
CYP4A38	NM_001048017	15	16642343-16661130
CYP4X1	NM_001206933	15	16513887-16550383
CYP4A37	NM_001048025	15	16607728-16627396
CYP4A11	NM_001048034	15	16667046-16677697
MYCBP	NM_001252277	15	6869338-6875102
COL9A2	NM_001197170	15	5651513-5665248
GUCA2A	NM_001198788	15	4091845-4093014
C15H16orf87	NM_001252134	15	11301397-11362176
AKR1A1	NM_001252168	15	17797814-17814006
PRDX1	NM_001252165	15	17844080-17861079
COR11G5	NM_001256439	15	20519789-20520728
COR11G9	NM_001256458	15	20615008-20615953
EDN2	NM_001003002	15	4649695-4656002
TPO	NM_001003009	17	3751326-3787572
DLA-79	NM_001020810	18	44176692-44179855
CTSD	NM_001025621	18	49037635-49046652
MEN1	NM_001081508	18	55372068-55377066
INS	NM_001130093	18	49347097-49347975
ZDHHC5	NM_001048105	18	41563134-41583336
PAX6	NM_001097544	18	38667100-38692590
IGF2	NM_001195827	18	49319981-49324713
OR4S2	NM_001017519	18	44605625-44606642
H19	NR_027327	18	49228073-49230776
BAD	NM_001031820	18	55807913-55817398
RTN3	NM_001197049	18	56267811-56325894

Gene ID	Transcript ID	CFA	Position
FTH1	NM_001193656	18	57495561-57498003
CCS	NM_001194970	18	53773614-53784735
C18H11orf31	NM_001164506	18	41544227-41545825
ARHGAP1	NM_001083367	18	45851166-45864357
ESRRA	NM_001002936	18	55782336-55787322
TH	NM_001002966	18	49350177-49358344
BEST1	NM_001097545	18	57498215-57509683
CCND1	NM_001005757	18	51527952-51535734
GPR44	NM_001048107	18	58458901-58460104
MYBPC3	NM_001048106	18	45305510-45322540
CFOLF1	NM_001013420	18	43031881-43032817
PGA	NM_001003117	18	58161063-58169739
PPP1CA	NM_001003064	18	53088537-53091233
SLC35C1	NM_001197189	18	46638965-46643653
OTUB1	NM_001251947	18	56060401-56067490
GSTP1	NM_001252167	18	52931172-52934194
NDUFS8	NM_001252295	18	52822422-52826817
COX8A	NM_001252370	18	56074916-56076543
COR12E4	NM_001256446	18	42977976-42978942
COR5L1	NM_001256459	18	43149323-43150259
EMR4P	NM_001038665	20	56287226-56322401
CCR1	NM_001038606	20	45423832-45424900
GNA11	NM_001195842	20	59126680-59146268
GNAI2	NM_001003364	20	42075070-42094683
ICAM1	NM_001003291	20	53821742-53830942
RTBDN	NM_001024961	20	52336436-52339536
PTH1R	NM_001003155	20	44904288-44923736
PRKCD	NM_001008716	20	39719805-39733242
CCR3	NM_001005261	20	45382085-45383165
CCL25	NM_001005259	20	55720277-55723000
CDC25A	NM_001145215	20	43822867-43846554
MADCAM1	NM_001024639	20	61123353-61127159
FUT5	NM_001005378	20	57153054-57154107
SLC1A6	NM_001003137	20	50101931-50147431
CD209	NM_001130832	20	55519911-55522762
STXBP2	NM_001003216	20	55415021-55423348
MAP2K2	NM_001048136	20	58411313-58433052
EPOR	NM_001048111	20	52947203-52952283
UBA52	NM_001128095	20	47479131-47481952
EMR3	NM_001038666	20	51022935-51062184

Gene ID	Transcript ID	CFA	Position
TJP3	NM_001003202	20	58785088-58811246
NICN1	NM_001033995	20	42809978-42813641
LSM4	NM_001110805	20	47709291-47722322
P2RY11	NM_001204441	20	53949065-53951664
PPAN	NM_001204439	20	53952165-53955871
DAG1	NM_001033992	20	42707986-42778256
CAPS	NM_001003282	20	57120209-57122104
CCR5	NM_001012342	20	45293946-45296903
CTXN1	NM_001039054	20	55614552-55615398
RAB8A	NM_001003152	20	49337460-49354767
AMT	NM_001033993	20	42815100-42819814
CFOLF4	NM_001013422	20	50503457-50504387
CD97	NM_001048110	20	51206780-51221467
GPX1	NM_001115119	20	42888451-42889540
AKAP8	NM_001007123	20	49802556-49819037
ELANE	NM_001003378	20	60930366-60932079
EMR2	NM_001038667	20	50932346-50971515
EMR1	NM_001038668	20	56349811-56410980
CAMP	NM_001003359	20	43789966-43791925
RHOA	NM_001003273	20	42823861-42886502
TLR9	NM_001002998	20	40537918-40542749
GNAT1	NM_001003068	20	42129454-42133249
COL7A1	NM_001002980	20	43518605-43549102
OAZ1	NM_001127234	20	59909662-59911267
INSL3	NM_001002962	20	48072720-48074039
MEF2B	NM_001205203	20	47016515-47020302
PRKACA	NM_001003032	20	51418218-51426727
PGR	NM_001003074	21	3446322-3551637
COL9A3	NM_001197171	24	49699827-49715611
ASIP	NM_001007263	24	26327359-26366307
DYNLRB1	NM_001252419	24	26605526-26625195
cOR9S7P	NR_003184	25	53115314-53115786
KIF1A	NM_001168473	25	53798855-53847130
MYEOV2	NM_001252368	25	53326485-53328909
COR9S5	NM_001256451	25	53233923-53234874
ATP2A2	NM_001003214	26	11167093-11227920
GPR81	NM_001145231	26	9751275-9752364
IL31	NM_001165914	26	10189892-10191937
DIABLO	NM_001079767	26	10141999-10160903
CDK2AP1	NM_001247976	26	9299518-9310043

**Gene ID**	**Transcript ID**	**CFA**	**Position**
P2RX7	NM_001113456	26	10959758-11002514
ADCY6	NM_001195147	27	8749358-8762733
KRT2	NM_001003386	27	5451448-5458087
KRT1	NM_001003392	27	5425744-5430755
SLC2A3	NM_001003308	27	40326321-40456764
KRT71	NM_001197029	27	5540425-5548698
AICDA	NM_001003380	27	40090203-40099888
LALBA	NM_001003129	27	8908492-8910507
KRT76	NM_001014277	27	5341863-5350185
NR4A1	NM_001003227	27	5949969-5957895
COR6C47	NM_001256498	27	3112606-3113542
PFDN5	NM_001251949	27	4910007-4913488
TMBIM6	NM_001252399	27	7930439-7953617
COR6C13	NM_001256457	27	3068399-3069338
CELA1	NM_001003007	27	6544238-6562635
INPP5A	NM_001003257	28	43443637-43558813
CYP2E1	NM_001003339	28	44089023-44099048
JMJD7	NM_001204235	30	11852230-11858678
SERF2	NM_001252267	30	13527896-13529647
CYP1A2	NM_001008720	30	40815980-40821668
SRP14	NM_001003251	30	10245557-10248171
UACA	NM_001003112	30	37374728-37420561
MAP2K1	NM_001048094	30	33677306-33753681
CLN6	NM_001011888	30	35239269-35257098
DUOX1	NM_001003122	30	14547768-14579947
RAD51	NM_001003043	30	10831576-10866143
NOX5	NM_001103218	30	35945947-35977553
SEC11C	NM_001003312	30	14327222-14327360
SMAD3	NM_001170829	30	34239835-34353623
RAB11A	NM_001003276	30	33195422-33213004
PLA2G4B	NM_001204236	30	11861238-11869210
RPL4	NM_001252409	30	33761011-33765929
ANP32A	NM_001003013	30	35736280-35773330
CRYAA	NM_001080898	31	39638621-39641814
TFF1	NM_001002992	31	39007462-39010556
TFF3	NM_001002990	31	38964755-38967690
TFF2	NM_001002991	31	38991640-38994417
POU1F1	NM_001006949	31	3768604-3786744
CXCL10	NM_001010949	32	3562713-3564233
PDE5A	NM_001003188	32	41197217-41327197

**Gene ID**	**Transcript ID**	**CFA**	**Position**
TFRC	NM_001003111	33	32248853-32270631
FYTTD1	NM_001251944	33	31960395-31982864
ABCB11	NM_001143932	36	16761407-16838078
GAD1	NM_001097543	36	18369305-18412283
DSTYK	NM_001025268	38	4669575-4716262
REN	NM_001003194	38	3744593-3754876
EIF2D	NM_001202488	38	5851832-5871373
CDC73	NM_001081507	38	8826027-8937749
OPTC	NM_001003056	38	3215294-3223138
PGRMC1	NM_001195148	X	94396259-94404968
USP11	NM_001197040	X	40774160-40790213
CD40LG	NM_001002981	X	110079649-110091767
CLIC2	NM_001195154	X	126283951-126306552
TIMP1	NM_001003182	X	41149215-41152786
PLP1	NM_001013834	X	80267541-80272547
VMA21	NM_001252312	X	122472261-122478087
F9	NM_001003323	X	112569285-112601743
BGN	NM_001003229	X	124373694-124377951
SSR4	NM_001204756	X	124652483-124657230
CXCR3	NM_001011887	X	58808308-58810581
MAOA	NM_001002969	X	37627025-37692688
IL13RA2	NM_001003075	X	90812306-90841873
AVPR2	NM_001003177	X	124796176-124798000
IDS	NM_001048125	X	120565481-120585912
MAOB	NM_001002970	X	37713740-37831886
EDA	NM_001014770	X	57008109-57439951
KDM5C	NM_001048032	X	44915435-44945394
KDM6A	NM_001197185	X	38693528-38907457
F8	NM_001003212	X	125917393-126063525
IL2RG	NM_001003201	X	58407336-58410934
CCNB3	NM_001005763	X	42916655-42965575
FOXP3	NM_001168461	X	42297698-42304349
COL4A5	NM_001002979	X	85051677-85316869
CLDN2	NM_001003089	X	83550925-83551878
HTR2C	NM_001006648	X	90541823-90703513
GDI1	NM_001003185	X	125185763-125190855
OPN1LW	NM_001197072	X	125018300-125031166
HPRT1	NM_001003357	X	108177628-108214917
FUNDC2	NM_001033512	X	126066788-126084918
SLC35A2	NM_001003059	X	42012029-42020803

**Gene ID**	**Transcript ID**	**CFA**	**Position**
AR	NM_001003053	X	54897733-55079812
EIF4E2	NM_001251943	X	95512628-95513587
BEX4	NM_001252047	X	79650618-79651205
RPS4X	NM_001252042	X	59216236-59221589
TIMM8A	NM_001252367	X	78333410-78336262
LOC403555	NM_001003031	X	94934017-94934910
