# Supplementary material for: Canine Mammary Tumours Are Affected by Frequent Copy Number Aberrations, including Amplification of MYC and Loss of PTEN
Source: PLoS One. 2015 May 8;10(5):e0126371. doi: 10.1371/journal.pone.0126371 (PMC4425491; doi:10.1371/journal.pone.0126371)
Supplement: S2 Table — All genes identified by PCF in the regions of loss found in ≥20% of the tumour samples. (PDF) [file pone.0126371.s002.pdf]

Supplementary file 1. Genes identified by PCF in recurrently gained regions.

All genes identified by PCF in the regions of gains found in ≥20% of the tumour samples.

Gene ID	Transcript ID	CFA	Position
HRH2	NM_001195844	4	40576920-40578000
NKX2-5	NM_001010959	4	42435898-42438988
GPX3	NM_001164454	4	61403963-61412303
KCNMA1	NM_001003300	4	30054840-30548254
RPS24	NM_001252318	4	31096902-31103176
KCNMB1	NM_001003299	4	44738415-44744170
PDE6A	NM_001003073	4	62307500-62367393
PDGFRB	NM_001003382	4	62129886-62165819
ADRB2	NM_001003234	4	63252138-63254084
ATOX1	NM_001003119	4	60803405-60812281
ARSI	NM_001048118	4	61991530-61996932
IL12B	NM_001003292	4	54386266-54396284
ADRA1B	NM_001197035	4	53879551-53927735
ATP6V0E1	NM_001003128	4	42569463-42601425
HAVCR1	NM_001205114	4	56249952-56266985
MSX2	NM_001003098	4	41194742-41199426
TCOF1	NM_001003057	4	61902458-61942067
NPM1	NM_001252171	4	43951435-43965146
HAVCR2	NM_001254715	4	56221919-56234965
MED7	NM_001253899	4	56201677-56206992
LPHN2	NM_001190488	6	68657023-69276367
UOX	NM_001011886	6	66541425-66572580
RPE65	NM_001003176	6	79953980-79977715
ABCA4	NM_001003360	6	58112924-58307873
FPGT	NM_001204810	6	75194919-75201096
SEP15	NM_001114760	6	64358655-64400381
PTGFR	NM_001048097	6	71519438-71558125
TNP2	NM_001013418	6	34541323-34542650
F3	NM_001024640	6	57764298-57775236
TNNI3K	NM_001204812	6	74888181-75170051
CRYZ	NM_001252397	6	74718508-74738016
PTGER3	NM_001002958	6	77997100-78050886
ASB17	NM_001003018	6	73787417-73804547
DIO2	NM_001122645	8	55723265-55732281
TNC	NM_001195149	11	72107499-72171280
STOM	NM_001142670	11	77297769-77311639

Gene ID	Transcript ID	CFA	Position
TLR4	NM_001002950	11	74385368-74396145
MYC	NM_001003246	13	28238007-28242545
TG	NM_001048104	13	32387064-32634247
ANXA13	NM_001003255	13	24996996-25047115
TRIB2	NM_001003218	17	12199316-12222005
SLC1A2	NM_001003138	18	35585196-35728282
CD44	NM_001197022	18	35757519-35846096
CXCR4	NM_001048026	19	41893757-41896848
EOGT	NM_001009187	20	25722634-25752975
FHIT	NM_001142257	20	33335946-34108777
MITF	NM_001003337	20	24853656-24884775
SELK	NM_001114878	20	39104869-39111567
CCDC66	NM_001168012	20	36711650-36756151
LOC476890	NM_001195607	21	47618245-47631703
SVIP	NM_001195609	21	47599254-47606525
BDNF	NM_001002975	21	51385774-51386908
MS4A1	NM_001048028	21	53902026-53913462
GIF	NM_001005759	21	53514534-53530364
MS4A2	NM_001003172	21	53629789-53636551
ANO5	NM_001168413	21	47104424-47175116
SLC17A6	NM_001168414	21	47206673-47245387
EDNRB	NM_001010943	22	34363950-34383792
CLN5	NM_001011556	22	33515050-33522369
THBD	NM_001006953	24	3597325-3599202
RRBP1	NM_001003179	24	8348115-8389374
SNRPB2	NM_001251968	24	9104830-9115983
FGFR2	NM_001003336	28	34303543-34406406
PNLIPRP1	NM_001003319	28	30168809-30184160
ADRB1	NM_001008713	28	27908600-27910061
MGMT	NM_001003376	28	41202021-41424823
PRDX3	NM_001256485	28	32396180-32404333
MCMBP	NM_001270851	28	32948279-32997174
BCL6	NM_001195404	34	23121696-23133055
TNFSF10	NM_001130836	34	39725658-39741399
IL12A	NM_001003293	34	29134783-29142272
GHSR	NM_001099945	34	39697541-39700712
SST	NM_001003307	34	23071464-23072894
FCER1A	NM_001110766	38	25696406-25701694
CD1A6	NM_001128837	38	26395315-26398237
CD1C	NM_001128836	38	26341099-26344187

Gene ID	Transcript ID	CFA	Position
SLAMF1	NM_001003084	38	24653358-24684966
CD1B	NM_001130830	38	26329119-26332684
FCER1G	NM_001003171	38	24267571-24270495
MGST3	NM_001252410	38	20669949-20691443
